# Editorial: Innovative integrated immuno—and inflammopharmacology in reference to autoimmune skin diseases

**DOI:** 10.3389/fcell.2023.1244332

**Published:** 2023-08-02

**Authors:** Magdalena Gabig-Cimińska, Celso Alves

**Affiliations:** ^1^ Department of Medical Biology and Genetics, University of Gdańsk, Gdańsk, Poland; ^2^ MARE—Marine and Environmental Sciences Centre/ARNET-Aquatic Research Network/ESTM, Polytechnic of Leiria, Peniche, Portugal

**Keywords:** autoimmunity, autoinflammation, skin diseases, molecular treatment, pharmacological therapy

Research in pharmacology for immune-mediated inflammatory skin diseases is advancing rapidly and has progressed beyond initial expectations in recent years. Indeed, extensive studies with innovative and rigorous methodologies that have deepened our understanding of the underlying mechanisms and pathophysiology of these conditions, led to the development of effective pharmacological interventions. This knowledge has enabled the development of a broad range of therapies that target various aspects of the disease process, leading to improved treatment options for patients. What is even more highly anticipated, however, is the identification—at the level of individual cells—of molecular patterns and trajectories of pharmaceutical particles with bridged immunoregulatory and anti-inflammatory effects in the affected body. Therapeutic cross-manipulations of autoimmune and anti-inflammatory responses can be achieved by both the immunoregulatory and anti-inflammatory effects of various innovative compounds, including small-molecule drugs, vaccines and biologics. The goal of this Research Topic was focused in the feature cutting-edge, high-resolution research describing the signature of pharmaceutical molecules in a single cell, related to the regulation of immune-inflammatory axis distortion of human diseases.

The collection gathered five articles regarding immune system regulation by pharmaceuticals as central to the outcomes of the intended applications, having contributions of scientists in the fields of molecular and medicinal biology, as well as pharmacology.

The case report titled “*Scleromyxedema associated with a monoclonal gammapathy: Successful treatment with intravenous immunoglobulins*” by Wang et al. described the successful treatment of a 45-year-old woman diagnosed with scleromyxedema associated with a monoclonal gammopathy, also known as paraproteinemia (Wang et al.). The disease refers to the co-occurrence of scleromyxedema, a skin disorder, and a monoclonal gammopathy, which involves the presence of abnormal proteins in the blood. In the referred case, the patient initially did not respond to systemic steroids, retinoids, and thalidomide, but showed significant improvement in both systemic and cutaneous symptoms after receiving treatment with high-dose intravenous immunoglobulin (IVIG). The case highlights the potential efficacy of IVIG, either as a standalone treatment or in combination with systemic steroids and/or thalidomide, as a first-line therapy for scleromyxedema associated with paraproteinemia. This approach proved successful in this particular patient, who had previously failed to respond to other treatments such as steroids, retinoids, and thalidomide. It is worth noting that this case report represents a single patient’s experience, and further research and clinical studies are needed to establish the broader effectiveness and safety of IVIG in the treatment of scleromyxedema associated with paraproteinemia. Nonetheless, this case report provides valuable information suggesting that IVIG may be a beneficial treatment option for patients who do not respond to other therapies.

Among biological processes, inflammation plays a critical role in the development of the immuno- and inflammopharmacology autoimmune skin diseases, being a consequence of the presence of immunocytes with overactive phenotype, the release of cytokines and the abnormal secretion of autoantibodies, leading to tissues and organs damage. Accordingly, Ben Abdallah et al. in their original article entitled “*Heat shock protein 90 inhibitor RGRN-305 potently attenuates skin inflammation*” explored the potential of HSP90 inhibition as a novel therapeutic approach for immune-mediated inflammatory skin diseases (Ben Abdallah et al.). The study investigates the anti-inflammatory effects and mechanisms of HSP90 inhibition using a specific inhibitor called RGRN-305 in models of inflammation induced by 12-O-tetradecanoylphorbol-13-acetate (TPA). The researchers conducted experiments using primary human keratinocytes stimulated with TPA and an irritative dermatitis murine model. They assessed the gene expression of proinflammatory markers using a Nanostring^®^ nCounter gene expression assay and quantitative real-time polymerase chain reaction (qRT-PCR). They also evaluated the efficacy of topical administration of RGRN-305 in reducing TPA-induced skin inflammation in mice, measuring ear thickness as an indicator of inflammation. The results showed that HSP90 inhibition with RGRN-305 significantly suppressed the expression of proinflammatory genes such as *TNF*, *IL1B*, *IL6*, and *CXCL8* in human keratinocytes. Topical application of RGRN-305 effectively reduced TPA-induced skin inflammation in mice, with a significant decrease in ear thickness. Gene expression analysis of ear tissue confirmed the reduction of proinflammatory markers. Furthermore, RNA sequencing revealed that RGRN-305 mitigated TPA-induced changes in gene expression and suppressed genes associated with inflammation. The study also found that the anti-inflammatory effects were mediated, at least partially, by inhibiting the activity of NF-kB, ERK1/2, p38 MAPK, and c-Jun signaling pathways. The findings suggest that HSP90 inhibition with RGRN-305 holds promise as a potential treatment for immune-mediated inflammatory skin diseases beyond psoriasis. The study highlights the robust suppression of inflammation by targeting key proinflammatory cytokines and signaling pathways. Moreover, the feasibility of topical administration of RGRN-305 is demonstrated, offering potential advantages such as reduced systemic exposure and improved tolerability. The research encourages further clinical evaluation of RGRN-305 in the treatment of inflammatory skin diseases, considering its multiple anti-inflammatory mechanisms and topical treatment potential.

Among autoimmune and autoinflammatory skin disorders, psoriasis a chronic, inflammatory, debilitating, and systemic disease, has a prevalence rate around 2%–3% in World population. However, the therapeutic approaches are limited. Gao et al. in their contribution “*Comparation of time-course, dose-effect, influencing factors and adverse events of biologics in the treatment of adults with moderate to severe plaque psoriasis*” evaluated the relative efficacy and safety of biologics used in the treatment of moderate to severe plaque psoriasis (MSPP), a significant advancement in dermatology therapeutics (Gao et al.). The study employs comparative effectiveness analysis, measuring the efficacy of various biological treatments for MSPP using Psoriasis Area and Severity Index (PASI) 75, 90, and 100 scores. The researchers utilize random models and a Bayesian method to compare the direct and indirect adverse events (AEs) of biologics with a placebo, providing probabilistic statements and predictions regarding their AEs. The dataset consists of summarized data from 54 trials, including 27,808 patients, involving the treatment of 17 different biologics. Three mathematical models are established to characterize the longitudinal profile of efficacy measures using nonparametric placebo evaluations. The results of the study indicate significant differences among the treatments. Bimekizumab, and ixekizumab emerge as the most effective treatments among the biologics studied, while the efficacy of sonelokimab requires further verification through additional studies. Additionally, ixekizumab and risankizumab exhibit relative stability and balanced performance in terms of efficacy and safety. The findings of this research provide valuable insights into the comparative effectiveness and safety of biologics in the treatment of MSPP. The results can aid in clinical decision-making and potentially improve patient outcomes. The impact of covariates, such as patients’ characteristics and treatment history, on drug efficacy is highlighted. On the other hand, Kuczyńska et al. in their revision entitled “*Molecular treatment trajectories within psoriatic T lymphocytes: a mini review*” identified the biological processes involved in the development and progression of psoriasis, a chronic immune-mediated inflammatory disease (IMID) (Kuczyńska et al.). The review highlights the molecular cascades that contribute to the pathological reactions occurring in both the affected skin and systemic compartments of individuals with psoriasis. The key players in these processes include local skin-resident cells derived from peripheral blood and skin-infiltrating cells, particularly T lymphocytes (T cells). The review emphasizes the interplay between the molecular components of T cell signaling transduction and their involvement in cellular cascades. Specifically, the Ca^2+^/CaN/NFAT, MAPK/JNK, PI3K/Akt/mTOR, and JAK/STAT pathways are discussed as important contributors to psoriasis pathogenesis. Despite the accumulating evidence identifying these pathways as potential targets for psoriasis management, their characterization and understanding are still not as comprehensive as desired. The review suggests that innovative therapeutic strategies utilizing synthetic Small Molecule Drugs (SMDs) and their combinations have shown promise in the treatment of psoriasis. These SMDs act on specific isoforms of pathway factors or individual effectors within T cells, aiming for incomplete blocking or modulation of disease-associated molecular pathways. While recent drug development has predominantly focused on biological therapies for psoriasis, which have limitations, SMDs targeting specific pathway factors or effectors within T cells could offer a valuable innovation in real-world treatment approaches for psoriasis patients. Overall, this review provides insights into the molecular processes involved in psoriasis pathogenesis and highlights the potential of SMDs as a promising approach in the treatment of psoriasis.

Furhtermore, Mao et al. in their paper “*The evaluation of JAK inhibitors on effect and safety in alopecia areata: a systematic review and meta-analysis of 2018 patients*” focused on the evaluation of the efficacy and safety of JAK inhibitors specifically in the context of alopecia areata (Mao et al.). The authors conducted a systematic review and meta-analysis of eligible studies retrieved from databases such as PubMed, Embase, Web of Science, and Clinical Trials. The search included studies published up to 30 May 2022, encompassing randomized controlled trials and observational studies that explored the use of JAK inhibitors for alopecia areata. The findings of the systematic review and meta-analysis suggest that JAK inhibitors are effective in treating alopecia areata. However, their use is associated with an increased risk of adverse effects. The researchers emphasize that the adverse effects observed were manageable within reasonable limits. Despite the positive results, the authors highlight the need for additional high-quality randomized controlled trials with larger sample sizes. These trials are crucial for identifying the ideal types and doses of JAK inhibitors that can optimize therapeutic efficiency while minimizing the potential harm associated with JAK suppression. Overall, this mini-review provides insights into the effectiveness and safety of oral JAK inhibitors in the treatment of alopecia areata. It underscores the importance of further research to refine the use of JAK inhibitors in this context.

Editors of this Research Topic thank all submitting authors for their work, reviewers for the time spent in assigning reviews, commenting on submitted manuscripts as well as reviewing. We hope that the articles presented herein will be a valuable resource for the community and help inspire future experimental innovations.

